# Supplementary material for: Heat adaptation of phage T7 under an extended genetic code
Source: Virus Evol. 2021 Dec 1;7(2):veab100. doi: 10.1093/ve/veab100 (PMC8923235; doi:10.1093/ve/veab100)
Supplement: veab100_Supp [file veab100_supp.zip › Sequencing_Analysis.zip!/Sequencing_Analysis/S1/evidence/JC_SIDE_2_60.html]

BRESEQ :: Evidence


| New junction evidence | | | | | | | | | | | |
| --- | --- | --- | --- | --- | --- | --- | --- | --- | --- | --- | --- |
|  | | seq id | position | reads (cov) | reads (cov) | score | skew | freq | annotation | gene | product |
| \* | ? | NC\_001604.1 | = 19635 | 4193 (1.000) | 12 (0.000) | 11/590 | 44.2 | 0.29% | intergenic (–/–) | *–/–* | –/– |
|  | ? | NC\_001604.1 | 19645 = | 4138 (1.000) |  |  |  |  | intergenic (–/–) | *–/–* | –/– |
| Rejected: Coverage evenness skew score above cutoff. | | | | | | | | | | | |
| Rejected: Frequency below/above cutoff threshold. | | | | | | | | | | | |

Only 100 of 4278 total aligned reads displayed.  

`CATTCACCACAAAGGAGACATTCTGGATGTAACGTCCGTTGTCATTAATCCTGAGGCAGACTCTAAGGGCTTACAGCGATTCCTAGCGAAACGCTTTAAGTACCTTGCGGAACTCCACGATTGCGATTGGGTGTCTCGTTGTAAGCATGAAGGCGAGACAATGCGTGTATACTTTAAGGAGGTATAAGTTATGGGTAAGAAAGTTAAGAAGGCCGTGAAGAAAGTCACCAAGTCCGTTAAGAAAGTCGTTAAGGAAGGGGCTCGTCCGGTTAAACAGGTTGCTGGCGGTCTAGCTGGTCTGGCTGGTGGTACTGGTGAAGCACAGATGGTGGAAGTACCACAAGCTGCCGCACAGATTGTTGACGTACCTGAGAAAGAGGTTTCCACTGAGGACGAAGCACAGACAGAAAGCGGACGCAAGAAAGCTCGTGCTGGCGGTAAGAAATCCTTGAGTGTAGCCCGTAGCTCCGGTGGCGGTATCAACATTTAATCAGGAGGTTATCGTGGAAGACTGCATTGAATGGACCGGAGGTGTCAACTCTAAGGGTTATGGTCGTAAGTGGGTTAATGGTAAACTTGTGACTCCACATAGGCACATCTAT  >  NC_001604.1/19345‑19946`  

                                                                                                                                                                                                                                                                                                            `|`                                                                                                                                                                                                                                                                                                               
        `acaAAGGAGACATTCTGGATGTAACGTCCGTTGTCATTAATCCTGAGGCAGACTCTAAGGGCTTACAGCGATTCCTAGCGAAACGCTTTAAGTACCTTGCGGAACTCCACGATTGCGATTGGGTGTCTCGTTGTAAGCATGAAGGCGAGACAATGCGTGTATACTTTAAGGAGGTATAAGTTATGGGTAAGAAAGTTAAGAAGGCCGTGAAGAAAGTCACCAAGTCCGTTAAGAAAGTCGTTAAGGAAGGGGCTCGTCCGGTTAAACAGGTTGCTGGCGGTCTAGCTGGTCTGGCtggtgg                                                                                                                                                                                                                                                                                                       <  1:312898/301‑1 (MQ=255)`  
        `acaAAGGAGACATTCTGGATGTAACGTCCGTTGACATTAATCCTGAGGCAGACTCTAAGGGCTTACAGCGATTCCTAGCGAAACGCTTTAAGTACCTTGCGGAACTCCACGATTGCGATTGGGTGTCTCGTTGTAAGCATGAAGGCGAGACAATGCGTGTATACTTTAAGGAGGTATAAGTTATGGGTAAGAAAGTTAAGAAGGCCGTGAAGAAAGTCACCAAGTCCGTTAAGAAAGTCGTTAAGGAAGGGGCTCGTCCGGTTAAACAGGTTGCTGGCGGTCTAGCTGGTCTGGCtggtgg                                                                                                                                                                                                                                                                                                       <  1:525883/301‑1 (MQ=255)`  
          `aaaGGAGACATTCTGGATGTAACGTCCGTTGTCATGAATCCTGAGGCAGACTCTAAGGGCTTACAGCGATTCCTAGCGAAACGCTTTAAGTACCTTGCGGAACTCCACGATTGCGATTGGGTGTCTCTTTGTAAGCATGAAGGCGAGACAATGCGTGTATACTTTAAGGAGGTATAAGTTATGGGTAAGAAAGTTAAGAAGGCCGTGAAGAAAGTCACCAAGTCCGTTAAGAAAGTCGTTAAGGAAGGGGCTCGTCCGGTTAAACAGGTTGCTGGCGGTCTAGCTGGTCTGGCTGGTGGTa                                                                                                                                                                                                                                                                                                     <  1:44859/301‑1 (MQ=255)`  
              `gagaCATTCTGGATGTAACGTCCGTTGTCATTAATCCTGAGGCAGACTCTAAGGGCTTACAGCGATTCCTAGCGAAACGCTTTAAGTACCTTGCGGAACTCCACGATTGCGATTGGGTGTCTCGTTGTAAGCATGAAGGCGAGACAATGCGTGTATACTTTAAGGAGGTATAAGTTATGGGTAAGAAAGTTAAGAAGGCCGTGAAGAAAGTCACCAAGTCCGTTAAGAAAGTCGTTAAGGAAGGGGCTCGTCCGGTTAAACAGGTTGCTGGCGGTCTAGCTGGTCTGGCTGGTGGTACTgg                                                                                                                                                                                                                                                                                                 <  1:204917/301‑1 (MQ=255)`  
              `gagaCATTCTGGATGTAACGTCCGTTGTCATTAATCCTGAGGCAGACTCTAAGGGCTTACAGCGATTCCTAGCGAAACGCTTTAAGTACCTTGCGGAACTCCACGATTGCGATTGGGTGTCTCGTTGTAAGCATGAAGGCGAGACAATGCGTGTATACTTTAAGGAGGTATAAGTTATGGGTAAGAAAGTTAAGAAGGCCGTGAAGAAAGTCACCAAGTCCGTTAAGAAAGTCGTTAAGGAAGGGGCTCGTCCGGTTAAACAGGTTGCTGGCGGTCTAGCTGGTCTGGCTGGTGGTACTgg                                                                                                                                                                                                                                                                                                 <  1:156963/301‑1 (MQ=255)`  
              `aaagagacaaggATGTAACGTCCGTTGTCATTAATCCTGAGGCAGACTCTAAGGGCTTACAGCGATTCCTAGGGAAACGCTTTAAGTACCTAGCGGAACTCCACGATTGCGATTGAGTGTATCGTTGTAAGCATGAAGGCGAGACAATGAGTGTATAATTTAAGGAGGTATAAGTTATGGGTAAGAAAGTTAAGAAGGCCGTGAAGAAAGTCACCAAGTCCGTTAAGAAAGTCGTTAAGGAAGGGGCTCGTCCGGTTAAACAGGTTGCTGGCGGTCTAGCTGGTCTGGCTGGTGGTACTgg                                                                                                                                                                                                                                                                                                 <  1:657814/291‑1 (MQ=255)`  
               `agaCATTCTGGATGTAACGTGCGGTGTCATAAATCTTGAGGCAGACTCTAAGGGCGTAAAGCGATTCCTAGCGAAACGCTTTAAGGACCTTGCGGAACTCCACGATTGCGATTGGGTGTCTCGTTGTAAGCATGAAGGCGAGACAATGCGTGTATACTTTAAGGAGGTATAAGTTATGGGTAAGAAAGTTAAGAAGGCAGTGAAGAAAGTCACCAAGTCCGTTAAGAAAGTCGTTAAGGAAGGGGCTCGTCCGGTTAAACAGGTTGCTGGCGGTCTAGCTGGTCTGGCTGGTGGTACTGGt                                                                                                                                                                                                                                                                                                <  1:383050/301‑1 (MQ=255)`  
                `aaaaatCTGGATTGAACGTCCGTTGTCATTAATCCTGAGGCAGACTCTAAGGGCTTACAGCGATTCCTAGCGAAACGCTTTAAGTACCTTGCGGAACTCCACGATTGCGATTGGGTGTCTCGTGGTAAGCATGAAGGCGAGACAATGCGTGTATACTTTAAGGAGGTATAAGTTATGGGTAAGAAAGTTAAGAAGGCCGTGAAGAAAGTCACCAAGTCCGTTACGAAAGTCGTTAAGGAAGGGGCTCGTCCGGTTAAACAGGTTGCTGGCGGTCTAGCTGGTCTGGCTGGTGGTACTGGTg                                                                                                                                                                                                                                                                                               <  1:628670/296‑1 (MQ=255)`  
                    `ttCTGGATGTAACGTCCGTTGTCATTAATCCTGAGGCAGACTCTAAGGGCTTACAGCGATTCCTAGCGAAACGCTTTAAGTACCTTGCGGAACTCCACGATTGCGATTGGGTGTCTCGTTGTAAGCATGAAGGCGAGACAATGCGTGTATACTTTAAGGAGGTATAAGTTATGGGTAAGAAAGTTAAGAAGGCCGTGAAGAAAGTCACCAAGTCCGTTAAGAAAGTCGTTAAGGAAGGGGCTCGTCCGGTTAAACAGTTTGCTGGCGGTCTAGCTGGTCTGGCTGGTGGTACTGGTgctgt                                                                                                                                                                                                                                                                                           >  1:532151/1‑297 (MQ=255)`  
                      `ttGGATGTAACGTCCGTTGTCATTAATCCTGAGGCAGACTCTAAGGGCTTACAGCGATTCCTAGCGAAACGCTTTAAGTACCTTGCGGAACTCCACGATTGCGATTGGGTGTCTCGTTGTAAGCATGAAGGCGAGACAATGCGTGTATACTTTAAGGAGGTATAAGTTATGGGTAAGAAAGTTAAGAAGGCCGTGAAGAAAGTCACCAAGTCCGTTAAGAAAGTCGTTAAGGAAGGGGCTCGTCCGGTTAAACAGGTTGCTGGCGGTCTAGCTGGTCTGGCTGGTGGTACTGGTGAAGcac                                                                                                                                                                                                                                                                                         <  1:235432/300‑1 (MQ=255)`  
                         `aaTGTAACGTCCGTTGTAATTAACCCTGAGGCAGACTCTAAGGGCTTACCGCGATTTCTAGCCAAACGCTGTAAGTACCTTGCGGAACTCCACTATTGCGATTGGGTGTCTCGTTGTAAGCATGAAGGCGAGACAATGCGTGTATTCTTTAAGGAGGTATAAGTTATGGGTAAGAAAGTTAAG‑AGGCCGTGAAGAAAGTCACCAAGTCCTTTAAGAAAGTCGTTAAGGAAGGGGCTCGTCCGGTTAAACAGGTTGCTGGCGGTCTAGCTGGTCTGGCTGGTGGTACTGGTGAAGCACAGAt                                                                                                                                                                                                                                                                                     <  1:597132/300‑1 (MQ=255)`  
                           `tGTAACGTCCGTTGTCATTAATCCTGAGGCAGACGCTAAGGGCTTACAGCGATTCCTAGCGAAACGATTGAAGTACCTTGCGGAACTCCACGATTGCGATTGGGTGTCTCGTTGTAAGCATGAAGGAGAGACAATGCGTGTATACTTTAAGGAGGTATAAGTTATGGGTAAGAAAGTTAAGAAGGACGTGAAGAAAGTCACCAAGTCCGTTAAGAAAGGCGTTAAGGAAGGGGCTCGTCCGGTTAAACAGGTTGCTGGCGGTCTAGCTGGTCTGGCTGGTGGTACTGGTGAAGCACAGAtg                                                                                                                                                                                                                                                                                    <  1:3655/301‑1 (MQ=255)`  
                           `tGTAACGTCCGTGGTCATTAATCCTGGGGCAGACTCTAAGGGCTTAAAGCGATTCCTAGCGAAACGCTTTAAGTACCTTGCGGAACTCCACGATTGCGATTGGGTGTCTCGTTGTAAGCATGAAGGCGAGACAATGCGTGTATACTTTAAGGAGGTATAAGTTATGGGTAAGAAAGTTAAGAAGGCCGTGAAGAAAGTCACCAAGTCCGTTAAGAAAGTCGTTAAGGAAGGGGCTCGTCCGGTTAAACAGGTTGCTGGCGGTCTAGCTGGTCTGGCTGGTGGTACTGGTGAAGCACAGAtg                                                                                                                                                                                                                                                                                    <  1:388767/301‑1 (MQ=255)`  
                                `ataagagacagaTTAATCCTGAGGCAGACTCTAAGGGCTTACAGCGATTCCTAGCGAAACGCTTTAAGTACCTTGCGGAACTCCACGATTGCGATTGGGTGTCTCGTTGTAAGCATGAAGGCGAGACAATGCGTGTATACTTTAAGGAGGTATAAGTTATGGGTAAGAAAGTTAAGAAGGCCGTGAAGAAAGTCACCAAGTCCGTTAAGAAAGTCGTTAAGGAAGGGGCTCGTCCGGTTAAACAGGTTGCTGGCGGTCTAGCTGGTCTGGCTGGTGGTACTGGTGAAGCACAGATGGTGGa                                                                                                                                                                                                                                                                               <  1:441346/290‑1 (MQ=255)`  
                                  `tgtataagagacagtCCTGAGGCAGACTCTAAGTGCTTACAGCGATTCCTAGCGAAACGCTTTAAGTACCTTGCGGAACTCCACGATTGCGATTGGGTGTCTCGTTGTAAGCATGAAGGCGAGACAATGCGTGTATACTTTAAGGAGGTATAAGTTATGGGTAAGAAAGTTAAGAAGGCCGTGAAGAAAGTCACCAAGTCCGTTAAGAAAGTCGTTAATGAAGGGGCTCGTCCGGTTAAACAGGTTGCTGGCGGTCTAGCTGGTCTGGCTGGTGGTACTGGTGAAGCACAGATGGTGGAAg                                                                                                                                                                                                                                                                             <  1:174652/287‑1 (MQ=255)`  
                                     `gTTGTCATTAATCCTGAGGCAGACTCTAAGGGCTTACAGCGATTCCTAGCGAAACGCTTTAAGTACCTTGCGGAACTCCACGATTGCGATTGGGTGTCTCGTTGTAAGCATGAAGGCGAGACAATGCGTGTATACTTTAAGGAGGTATAAGTTATGGGTAAGAAAGTTAAGAAGGCCGTGAAGAAAGTCACCAAGTCCGTTAAGAAAGTCGTTAAGGAAGGGGCTCGTCCGGTTAAACAGGTTGCTGGCGGTCTAGCTGGTCTGGCTGGTGGTACTGGTGAAGCACAGATGGTGGAAGTAc                                                                                                                                                                                                                                                                          >  1:26007/1‑301 (MQ=255)`  
                                        `gTCATTAATCCTGAGGCAGACTCTAAGGGCTTACAGCGATTCCTAGCGAAACGCTTTAAGTACCTTGCGGAACTCCACGATTGCGATTGGGTGTCTCGTTGTAAGCATGAAGGCGAGACAATGCGTGTATACTTTAAGGAGGTATAAGTTATGGGTAAGAAAGTTAAGAAGGCCGTGAAGAAAGTCACCAAGTCCGTTAAGAAAGTCGTTAAGGAAGGGGCTCGTCCGGTTAAACAGGTTGCTGGCGGTCTAGCTGGTCTGGCTGGTGGTCCTGGTGAAGCACAGATGGTGGAAGtgtgtc                                                                                                                                                                                                                                                                       >  1:83879/1‑296 (MQ=255)`  
                                                      `ggCAGACTCTAAGGGCTTACAGCGATTCCTAGCGAAACGCTTTAAGTACCTTGCGGAACTCCACGATTGCGATTGGGTGTCTCGTTGTAAGCATGAAGGCGAGACAATGCGTGTATACTTTAAGGAGGTATAAGTTAGGGGTAAGAAAGTTAAGAAGGCCGTGAAGAAAGTCACCAAGTCCGTTAAGAAAGTCGTTAGGGAAGGGGCTCGTCCGGTTAAACAGGTTGCTGGCGGTCTAGCTGGTCTGGCTGGTGGTACTGGTGAAGCACAGATGGTGGAAGTACCACAAGCTGCCGCACAg                                                                                                                                                                                                                                                         >  1:670350/1‑301 (MQ=255)`  
                                                          `gACTCTAAGGGCTTACAGCGATTCCTAGCGAAACGCTTTAAGTACCTTGCGGAACTCCACGATTGCGATTGGGTGTCTCGTTGTAAGCATGAAGGCGAGACAATGCGTGTATACTTTAAGGAGGTATAAGTTTTGGGTAAGAAAGTTAAGAAGGCCGTGAAGAAAGTCACCAAGTCCGTTAAGAAAGTCGTTAAGGAAGGGGCTCGTCCGGTTAAACCGGTTGCTGGCGGTCTAGCTGGTCTGGCTGGTGGTACTGGTGAAGCACAGATGGTGGAAGTACCACAAGCTGCCGCACAGAttg                                                                                                                                                                                                                                                     >  1:328862/1‑301 (MQ=255)`  
                                                          `gACTCTAAGGGCTTACAGCGATTCCTAGCGAAACGCTTTAAGTACCTTGCGGAACTCCACGATTGCGATTGGGTGTCTCGTTGTAAGCATGAAGGCGAGACAATGCGTGTATACTTTAAGGAGGTATAAGTTATGGGTAAGAAAGTTAAGAAGGCCGTGAAGAAAGTCACCAAGTCCGTTAAGAAAGTCGTTAAGGAAGGGGCTCGTCCGGTTAAACAGGTTGCTGGCGGTCTAGCTGGTCTGGCTGGTGGTACTGGTGAAGCACAGATGGTGGAAGTACCACAAGCTGCCGCCAAGAttg                                                                                                                                                                                                                                                     >  1:604066/1‑301 (MQ=255)`  
                                                            `ctctAAGGGCTTACAGCGATTCCTAGCGAAACGCTTTAAGTACCTTGCGGAACTCCACGATTGCGATTGGGTGTCTCGTTGTAAGCATGAAGGCGAGACAATGCGTGTATACTTTAAGGAGGTATAAGTTATGGGTAAGAAAGTTAAGAAGGCCGTGAAGAAAGTCACCAAGTCCGTTAAGAAAGTCGTTAAGGAAGGGGCTCGTCCGGTTAAACAGGTTGCTGGCTGTCTAGCTGGTCTGGCTGGTGGTACTGGTGAAGCACAGATGGTGGAAGTACCACAAGCTGCCGCACAGCttgtt                                                                                                                                                                                                                                                   >  1:47962/1‑301 (MQ=255)`  
                                                            `ctctAAGGGCTTACAGCGATTCCTAGCGAAACGCTTTAAGTACCTTGCGGAACTCCACGATTGCGATTGGGTGTCTCGTTGTAAGCATGAAGGCGAGACAATGCGTGTATACTTTAAGGAGGTATAAGTTATGGGTAAGAAAGTTAAGAAGGCCGTGAAGAAAGTCACCAAGTCCGTTAAGAAAGTCGTTAAGGAAGGGGCTCGTCCGGTTAAACAGGTTGCTGGCGGTCTAGCTGGTCTGGCTGGTGGTACTGGTGAAGCACAGATGGTGGAAGTACCACAAGCTGCCGCACAGAttgtt                                                                                                                                                                                                                                                   >  1:555053/1‑301 (MQ=255)`  
                                                            `ctctAAGGGCTTACAGCGATTCCTAGCGAAACGCTTTAAGTACCTTGCGGAACTCCACGATTGCGATTGGGTGTCTCGTTGTAAGCATGAAGGCGAGACAATGCGTGTATACTTTAAGGAGGTATAAGTTATGGGTAAGAAAGTTAAGAAGGCCGTGAAGAAAGTCACCAAGTCCGTTAAGAAAGTCGTTAAGGAAGGGGCTCGTCCGGTTAAACAGGTTGCTGGCGGTCTAGCTAGTCTGGCTGGTGGTACTGGTGAAGCACAGATGGTGGAAGTACCACAAGCTGCCGCACAGAttgtt                                                                                                                                                                                                                                                   >  1:562229/1‑301 (MQ=255)`  
                                                            `ctctAAGGGCTTACAGCGATTCCTAGCGAAACGCTTTAAGTACCTTGCGGAACTCCACGATTGCGATTGGGTGTCTCGTTGTAAGAATGAAGGCGAGACAATGCGTGTATACTTTAAGGAGGTATAAGTTATGGGTAAGAAAGTTAAGAAGGCCGTGAAGAAAGTCACCAAGTCCGTTAAGAAAGTCGTTAAGGAAGGGGCTCGTCCGGTTAAACAGGTTGCTTGCGGTCTAGCTGGTCTGGCTGGGGGTACTGGTGAAGCACAGATGGTGGACGTACCATAAGCTGCCGCAAAGAttgtt                                                                                                                                                                                                                                                   >  1:163047/1‑301 (MQ=255)`  
                                                                `aaGGGCTTACAGCGATTTCTAGCGAAACGCTTTAAGTACCTTGCGGAACTCCACGATTGCGATTGGGTGTCTCGTTGTAAGCATGAAGGCGAGACAATGCGTGTATACTTTAAGGAGGTATAAGGTATGGGTAAGAAAGTTAAGAAGGCCGTGAAGAAAGTCACCAAGTCCGTTAAGAAAGTCGTTAAGGAAGGGGCTCGTCCGGTTAAACAGGTTGCTGGCGGTCTAGCTGGTCTGGCTGGTGGTACTGGTGAAGCACAGATGGTGGAAGTACCACAAGCTGCCGCACAGATTGTTGACg                                                                                                                                                                                                                                               <  1:396374/301‑1 (MQ=255)`  
                                                                `aaGGGCTTACAGCGATTCCTAGCGAAACGCTTTAAGTACCTTGCGGAACTCCACGATTGCGATTGGGTGTCTCGTTGTAAGCATGAAGGCGAGACAATGCGTGTATACTTTAAGGAGGTATAAGTTATGGGTAAGAAAGTTAAGAAGGCCGTGAAGAAAGTCACCAAGTCCGTTAAGAAAGTCGTTAAGGAAGGGGCTCGTCCGGTTAAACAGGTTGCTGGCGGTCTAGCTGGTCTGGCTGGTGGTACTGGTGAAGCACAGATGGTGGAAGTACCACAAGCTGCCGCACAGATTGTTGACg                                                                                                                                                                                                                                               <  1:149436/301‑1 (MQ=255)`  
                                                                  `gggCTTACAGCGATTCCTAGCGAAACGCTTTAAGTACCTTGCGGAACTCCACGATTGCGATTGGGTGTCTCGTTATAAGCATGAAGGCGAGACAATGCGTGTATACTTTATGGAGGTATAAGTTATGGGTAAGAAAGTTAAGAAGGCCGTGAAGAAAGTCACCAAGTCCGTTAAGAAAGTCGTTAAGGAAGGGGCTCGTCCGGTTAAACAGGTTGCTGGCGGTCTAGCTGGTCTGGCTGGTGGTACTGGTGAAGCACAGATGGTGGAAGTACCACAAGCTGCCGCACAGATTGTTGACGTa                                                                                                                                                                                                                                             <  1:402117/301‑1 (MQ=255)`  
                                                                   `ggCTTACAGCGATTCCTAGCGAAACGCTTTAAGTACCTTGCGGAACTCCACGATAGCGATTGGGTGTCTCGTTGTAAGCATGAAGGCGAGACAAAGCGTGTAAACTTTAAGGTGGTATAAGCTATGGGTAAGAAAGTTAAGAAGGCCGTGAAGAAAGTCACCAAGACCGTTAAGAAAGTCGTTAAGGAAGGGGCTCGTCCGGTTAAACAGGTTGCTGGCGGTCGAGCTGGTCTGGCTGGTGGTACTGGTGAAGCACAGATGGTGGAAGTACCACAAGCTGCCGCACAGATTGTTGACGTAc                                                                                                                                                                                                                                            >  1:502549/1‑301 (MQ=255)`  
                                                                               `ttCCTAGGGAAACGGTTTAAGTACCTTGCGGAACTCCACGATTGCGATTGGGTGTCTCGTTGTAAGCATGAAGGCGAGACAATGCGTGTATACGTTAAGGAGGTATAAGTTATGGGTAAGAAAGTTAAGAAGGCCGTGAAGAAAGGCACCAAGTCCGTTAAGAAAGTCGTTAAGGAAGGGGCTCGTCCGGTTAAACAGGTTGCTGGCGGTCTAGCTGGTCTGGCTGGTGGTACTGGTGAAGCACAGATGGTGGAAGTACCACAAGCTGCCGCACAGATTGTTGACGTACCTGAGAAAGAgg                                                                                                                                                                                                                                <  1:301615/301‑1 (MQ=255)`  
                                                                               `ttCCTAGCGAAACGCTTTAAGTAACTGGCGGAAATCCACGATTGCGATTGGGTGTTTCGTTGTAAGCATGAAGGCGAGAAAAGGCGTGTATACTTTAAGGAGGTATAAGTTATGGGTAAGAAAGTGAAGAAGGCCGTGAAGAAAGTCACCAAGTCCGTTAAGAAAGTCGTTAAGGAAGGGGCTCGTCCGGTTAAACAGGTTGCTGGCGGTCTAGCTGGTCTGGCTGGTGGTACTGGTGAAGCACAGATGGTGGAAGTACCACAAGCTGCCGCACAGATTGTTGACGTACCTGAGAAAGAgg                                                                                                                                                                                                                                <  1:419650/301‑1 (MQ=255)`  
                                                                               `ttCCGAGCGAAACGCTTTAAGTACCTTGCGGAACTCCACGATTGCGATTGGGTGTCTCGTTGTAAGCATGAAGGCGAGACAATGCGTGTATACTTTAAGGAGGTATAAGTTATGGGTAAGAAAGTTAAGAAGGCCGTGAAGAAAGTCACCAAGTCCGTTAAGAAAGTCGTTAAGGAAGGGGCTCGTCCGGTTAAACAGGTTGCTGGCGGTCTAGCTGGTCTGGCTGGTGGTACTGGTGAAGCACAGATGGTGGAAGTACCACAAGCTGCCGCACAGATTGTTGACGTACCTGAGAAAGAgg                                                                                                                                                                                                                                <  1:489636/301‑1 (MQ=255)`  
                                                                                    `tataagagacagtAAGTACCTTGCGGAACTCCACGATTGCGATTGGGTGTCTCGTTGTAAGCATGAAGGCGAGACAATGCGTGTATACTTTAAGGAGGTATAAGTTATGGGTAAGAAAGTTAAGAAGGCCGTGAAGAAAGTCACCAAGTCCGTTAAGAAAGTCGTTAAGGAAGGGGCTCGTCCGGTTAAACAGGTTGCTGGCGGTCTAGCTGGTCTGGCTGGTGGTACTGGTGAAGCACAGATGGTGGAAGTACCACAAGCTGCCGCACAGATTGTTGACGTACCTGAGAAAGAGGTTTcc                                                                                                                                                                                                                           <  1:119347/289‑1 (MQ=255)`  
                                                                                             `cTTTAAGTACCTTGCGGAACTCCACGATTGCGATTGGGTGTCTCGTTGTAAGCATGAAGGCGAGACAATGCGTGTATACTTTAAGGAGGTATAAGTTATGGGTGAGAAAGTTAAGAAGGCCGTGAAGAAAGTCACCAAGTCCGTTAAGAAAGTCGTTAAGGAAGGGGCTCGTCCGGTTAAACAGGTTGCTGGCGGTCTAGCTGGTCTGGCTGGTGGTACTGGTGAAGCACAGATGGTGGAAGTACCACAAGCTGCCGCACAGATTGTTGACGTACCTGAGAAAGAGGTTTCCACTGAGGAc                                                                                                                                                                                                                  >  1:569449/1‑301 (MQ=255)`  
                                                                                             `cTTTAAGTACCTTGCGGAACTCCACGATTGCGATTGGGTGTCTCGTTGTAAGCATGAAGGCGAGACAATGCGTGTATACTTTAAGGAGGTATAAGTTATGGGTAAGAAAGTTAAGAAGGCCGTGAAGAAAGTCACCAAGTCCGTTAAGAAAGTCGTTAAGGAAGGGGCCCGTCCGGTTAAACAGGTTGCTGGCGGTCTAGCTGGTCTGGCTGGTGGTACTGGTGAAGCACAGATGGTGGAAGTACCACAAGCTGCCGCACAGATTGTTGACGTACCTGAGAAAGAggctgtctcttataca                                                                                                                                                                                                                  >  1:408642/1‑287 (MQ=255)`  
                                                                                               `ataaggaacgggaggAACACCACGAATGCGATTGGGTGTCTCGTTGTAAGCATGAAGGCGAGACAATGCGTGTATACTTTAAGGAGGTATAAGTTATGGGTAAGAAAGTTAAGAAGGCCGTGAAGAAAGTCACCAAGTCCGTTAAGAAAGTCGTTAAGGAAGGGGCTCGTCCGGTTAAACAGGTTGCTGGCGGTCTAGCTGGTCTGGCTGGTGGTACTGGTGAAGCACAGATGGTGGAAGTACCACAAGCTGCCGCACAGATTGTTGACGTACCTGAGAAAGAGGTTTCCACTGAGGACGa                                                                                                                                                                                                                <  1:265413/288‑1 (MQ=255)`  
                                                                                                  `cagaCCTGGCGGAACTCCACGAGTGCGATTGGGTGTCTCGTTGTAAGCATGAAGGCGAGACAATGCGTGTATACTTTAAGGAGGTATAAGTTATGGGTAAGAAAGTTAAGAAGGCCGTGAAGAAAGTCACCAAGTCCGTTAAGAAAGTCGTTAAGGAAGGGGCTCGTCCGGTTAAACAGGTTGCTGGCGGTCTAGCTGGTCTGGCTGGTGGTACTGGTGAAGCACAGATGGTGGAAGTACCACAAGCTGCCGCACAGATTGTTGACGTACCTGAGAAAGAGGTTTCCACTGAGGACGAAGc                                                                                                                                                                                                             <  1:197962/298‑1 (MQ=255)`  
                                                                                                  `aGTACCTTGCGGAACTCCACGATTGCGATTGGGTGTCTCGTTGTAAGCATGAAGGCGAGACAATGCGTGTATACTTTAAGGAGGTATAAGTTATGGGTAAGAAAGTTAAGAAGGCCGTGAAGAAAGTCACCAAGTCCGTTAAGAAAGTCGTTAAGGAAGGGGCTCGTCCGGTTAAACAGGTTGCTGGCGGTCTAGCTGGTCTGGCTGGTGGTACTGGTGAAGCACAGATGGTGGAAGTACCACAAGCTGCCGCACAGATTGTTGACGTACCTGAGAAAGAGGTTTCCACTGAGGACGAAGc                                                                                                                                                                                                             <  1:276334/301‑1 (MQ=255)`  
                                                                                                    `taaccttcGGAACTTCACGATTGCGATTTGGTGTCTCGTTGTAAGCATGAAGGCGAGACAATGCGTGGATACTTTAAGAAGGTATAAGTTATGGGTAAGAAAGTTAAGAAGGCCGTGAAGAAAGTCACCAAGTCCGTTAAGAAAGGCGTTAAGGAAGGGGCTCGTCCGGTTAAACAGGTTGCTGGCGGTCTAGCTGGTCTGGCTGGTGGTTCTGGTGAAGCACAGATGGTGGAAGTACCACAAGCTGCCGCACAGATTGTTGACGTACCTGAGAAAGAGGTTTCCACTGAGGACGAAGcac                                                                                                                                                                                                           <  1:641426/294‑1 (MQ=255)`  
                                                                                                              `aagtCCAAGAGTGCGATTGGGTGGCGAGGTGTAAGAATGAAGGGGGGACAATGCGGGGATACTTTAAGGAGGTAAAAGTTAAGGGTAAAAAAGTTAAGAAGGACGTGAAGAAAGTCACCAAGTCCGTTAAGAAAGGCGTTAAGGAAGGGGCTCGTCCGGTTAAACAGGTTGCTGGCGGTCTAGCTGGTCTGGCTGGTGGTACTGGTGAAGCACAGATGGTGGAAGTACCACAAGCTGCCGCACAGATTGTTGACGTACCTGAGAAAGAGGTTTCCACTGAGGACGAAGCACAGACAGAAAg                                                                                                                                                                                                 <  1:414620/298‑1 (MQ=255)`  
                                                                                                                `cTCCACGATTGCGATTGGGTGTCTCGTTGTAAGCATGAAGGCGAGACAATGCGTGTATACTTTAAGGAGGTATAAGTTATGGGTAAGAAAGTTAAGAAGGCCGTGAAGAAAGTCACCAAGTCCGTTAAGAAAGTCGTTAAGGAAGGGGCTCGTCCGGTTAAACAGGTTGCTGGCGGTCTAGCTGGTCTGGCTGGTGGTACTGGTGAAGCACAGATGGTGGAAGTACCACAAGCTGCCGCACAGATTGTTGTCGTACCTGAGAAAGAGGTTTCCACTGAGGACGAAGCACAGACAGAAAGCg                                                                                                                                                                                               >  1:495187/1‑301 (MQ=255)`  
                                                                                                                `cTCCACGATTGCGATTGGGTGTCTCGTTGTAAGCATGAAGGCGAGACAATGCGTGTATACTTTAAGGAGGTATAAGTTATGGGTAAGAAAGTTAAGAAGGCCGTGAAGAAAGTCACCAAGTCCGTTAAGAAAGTCGTTAAGGAAGGGGCTCGTCCGGTTAAACAGGTTGCTGGCGGTCTAGCTGGTCTGGCTGGTGGTACTGGTGAAGCACAGATGGTGGAAGTACCACAAGCTGCCGCACAGATTGTTGACGTACCTGAGAAAGAGGTTTCCACTGAGGACGAAGcacctgtctcttata                                                                                                                                                                                               >  1:138142/1‑289 (MQ=255)`  
                                                                                                                  `ccACGATTGCGATTGGGTGTCTCGTTGTAAGCATGAAGGCGAGACAATGCGTGTATACTTTAAGGAGGTATAAGTTATGGGTAAGAAAGTTAAGAAGGCCGTGAAGAAAGTCACCAAGTCCGTTAAGAAAGTCGTTAGGGAAGGGGCTCGTCCGGTTAAACAGGTTGCGGGCGGTCTAGCTGGTCTGGCTGGTGGTACTGGTGAAGCACAGATGGTGGAAGTACGACAAGCTGCCGCACAGATTGTTGACGTACCTGAGAAAGAGGTTTCCACTGAGGACGAAGCACAGACAGAAAGCGGa                                                                                                                                                                                             >  1:289202/1‑301 (MQ=255)`  
                                                                                                                             `attgGGTTTCTCTTTGTAAGAATGAAGGCGAAACAATTCGTGTATAAGTTAAGAGAGTATAAGTTGTGGGTAAGAAAGTTAAGAAGGCCGTGAAGAAAGTCACCTAGTCCGTTAAGAAAGTCGTTAAGGAAGGGGCTCGTCCGGTTAAACAGGTTGCTGGCGGTCTAGCTGGTATGGCTGGTGGTACTGGTGAAGCACAGATGGTGGAAGTACCACAAGCTGCCGCACAGATTGTTGACGTACCTGAGAAAGAGGTTTCCACTGAGGACGAAGCACAGACAGAAAGCGGACGCAAGAAAGc                                                                                                                                                                                  <  1:112327/301‑1 (MQ=255)`  
                                                                                                                                 `ggTGTCTCGTTGTAAGCATGAAGGCGAGACAATGCGTGTATACTTTAAGGAGGTATAAGTTATGGGTAAGAAAGTTAAGAAGGCCGTGAAGAAAGTCACCAAGTCCGTTAAGAAAGTCGTTAAGGAAGGGGCTCGTCCGGTTAAACAGGTTGCTGGCGGTCTAGATGGTCTGGCTGGTGGTACTGGTGAAGCACAGATGGTGGAAGTACCACAAGCTGCCGCACAGATTGTTGACGTACCTGAGAAAGAGGTTTCCACTGAGGACGAAGCACAGACAGAAAGCGGACGCAAGAAAGCTCGt                                                                                                                                                                              >  1:191596/1‑301 (MQ=255)`  
                                                                                                                                        `cGTTGTAAGCATGAAGGCGAGACAATGCGTGTATACTTTAAGGAGGTATAAGTTATGGGTAAGAAAGTTAAGAAGGCCGTGAAGAAAGTCACCAAGTCCGTTAAGAAAGTCGTTAAGGAAGGGGCTCGTCCGGTTAAACAGGTTGCTGGCGGTCTAGCTGGTCTGGCTGGTGGTACTGGTGAAGCACAGATGGTGGAAGTACCACAAGCTGCCGCACAGATTGTTGACGTACCTGAGAAAGAGGTTTCCACTGAGGACGAAGCACAGACAGAAAGCGGACGCAAGAAAGCTCGTGCTGGCg                                                                                                                                                                       >  1:78071/1‑301 (MQ=255)`  
                                                                                                                                         `gTTGTAAGCATGAAGGCGAGACAATGCGTGTATACTTTAAGGAGGTATAAGTTATGGGTAAGAAAGTTAAGAAGGCCGTGAAGAAAGTCACCAAGTCCGTTAAGAAAGTCGTTAAGGAAGGGGCTCGTCCGGTTAAACAGGTTGCTGGCGGTCTAGCTGTTCTGGCTGGTGGTACTGGTGAAGCACAGATGGTGGAAGTACCACAAGCTGCCGCACAGATTGTTAACGTACCTGAGAAAGAGGTTTCCACTTAGGACGAAGCACAGACAGAAAGCGGACGCAAGAAAGCTctgtctcttat                                                                                                                                                                      >  1:253140/1‑291 (MQ=255)`  
                                                                                                                                            `gTAAGCATGAAGGCGAGACAATGCGTGTATACTTTAAGGAGGTATAAGTTATGGGTAAGAAAGTTAAGAAGGCCGTGAAGAAAGTCACCAAGTCCGTTAAGAAAGTCGTTAAGGAAGGGGCTCGTCCGGTTAAACAGGTTGCTGGCGGTCTAGCTGGTCTGGCTGGTGGTACTGGTGAAGCACAGATGGTGGAAGTACCACAAGCTGCCGCACAGATTGTTGACGTACCTGAGAAAGAGGTTTCCACTGAGGACGAAGCACAGACAGAAAGCGGACGCAAGAAAGCTCGTGCTGGCGGTaa                                                                                                                                                                   >  1:537701/1‑301 (MQ=255)`  
                                                                                                                                             `tAAGCATGAAGGCGAGACAATGCGGGTATAATTTAAGGATGTATAAGGTATGGGTAAGAAAGGTAAGAAGGCCGTGAAGAAAGTCACCAAGTCCGTTAAGAAAGTCGTTAAGGAAGGGGCTCGTCCGGTTAAACAGGTGGCTGGCGGTCTAGCTGGTCTGGCTGGTGGTACTGGTGAAGCACAGATGGTGGAAGTACCACAAGCTGCCGCACAGATTGTTGACGTACCTGAGAAAGAGGTTTCCACTGAGGACGAAGCACAGACAGAAAGCGGACGCAAGAAAGCTCGTTCTGGCGGTAAg                                                                                                                                                                  <  1:186030/301‑1 (MQ=255)`  
                                                                                                                                                      `aGGAGAGACAATGCGTGTATACTTTAAGGAGGTATAAGTTGTGGGTCAGAAAGTTAAGCAGGCCGTGAAGAAAGTCACCAAGTCCGTTAAGAAAGTCGTTAAGGAAGGGGCTCGTCCGGTTAAACAGGTTGCTGGCGGTCTAGCTGGTCTGGCTGGTGGTACTGGTGAAGCACAGATGGTGGAAGTACCACAAGCTGCCGCACAGAGTGTTGACGTACCTGAGAAAGAGGTTTCCACTGAGGACGAAGCACAGACAGAAAGCGGACGCAAGAAAGCTCGTGCTGGCGGTAAGAAATCCTTg                                                                                                                                                         <  1:514493/301‑1 (MQ=255)`  
                                                                                                                                                           `agaCAATGCGTGTATACTTTAAGGAGGTATAAGTTATGGGTAAGAAAGTTAAGAAGGCCGTGAAGAAAGTCACCAAGTCCGTTAAGAAAGTCGTTAAGGAAGGGGCTCGTCCGGTTAAACAGGTTGCTGGCGGTCTAGCTGGTCTGGCTGGTGGTACTGGTGAAGCACAGATGGTGGAAGTACCACAAGCTGCCGCACAGATTGTTGACGTACCTGAGAAAGAGGTTTCCACTGAGGACGAAGCACAGACAGAAAGCGGACGCAAGAAAGCTCGTGCTGGCGGTAAGAAATCCTTGAgtgt                                                                                                                                                    <  1:583824/301‑1 (MQ=255)`  
                                                                                                                                                            `gaCAATGCGTGTATACTTTAAGGAGGTATAAGTTATGGGTAAGAAAGTTAAGAAGGCCGTGAAGAAAGTCATCAAGTCCGTTAAGAAAGTCGTTAAGGAAGGGGCTCGTCCGGTTAAACAGGTTGCTGGCGGTCTAGCTGGTCTGGCTGGTGGTACTGGTGAAGCACAGATGGTGGAAGTACCACAAGCTGCCGCACAGATTGTTGACGTACCTGAGAAAGAGGTTTCCACTGAGGACGAAGCACAGACAGAAAGCGGACGCAAGAAAGCTCGTGCTGGCGGTAAGAAATCCTTGAgtgtc                                                                                                                                                   >  1:358690/1‑300 (MQ=255)`  
                                                                                                                                                                  `gCGTGTATACTTTAAGGAGGTATAAGTTATGGGTAAGAAAGTTAAGAAGGCCGTGAAGAAAGTCACCAAGTCCGTTAAGAAAGTCGTTAAGGAAGGGGCTCGTCCGGTTAAACAGGTTGCTGGCGGTCTAGCTGGTCTGGCTGGTGGTACTGGTGAAGCACAGATGGTGGAAGTACCACAAGCTGCCGCACAGATTGTTGACGTACCTGAGAAAGAGGTTTCCACTGAGGACGAAGCACAGACAGAAAGCGGACGCAAGAAAGCTCGTGCTGGCGGTAAGAAATCCTTTCGTGTAGCCCGt                                                                                                                                             >  1:590499/1‑301 (MQ=255)`  
                                                                                                                                                                    `gtgtATACTTTAAGGAGGTATAAGTTATGGGTAAGAAAGTTAAGAAGGCCGTGAAGCAAGTCACCAAGTCCGCTAAGAAAGTCGTTAAGGAAGGGTCTCGTCCGGTTAAACAGGTTGCTGGCGGTCTAGCTGGTCTGTTTGGTGGTACTGGTGAAGCACAGATGGTGGAAGTACCACAAGCTGCCGCACAGATTGTTGACGTACTTGAGAAAGAGGTCTCCACTGAGGATGAAGCACAGACAGAAAGCGGACGTAAGAAAGCTCATGCTGGCGGTAAGAAACCCTTGAGTGTATCCCGTAg                                                                                                                                           >  1:543140/1‑301 (MQ=255)`  
                                                                                                                                                                    `gtgtATACTTTAAGGAGGTATAAGTTATGGGTAAGAAAGTTAAGAAGGCCGTGAAGAAAGTCACCAAGTCCGTTAAGAAAGTCGTTAAGGAAGGGGCTCGTCCGGTTAAACAGGTTGCTGGCGGTCTAGCTGGTCTGGCTGGTGGTACTGGTGAAGCACAGATGGTGGAAGTACCACAAGCTGCCGCACAGATTGTTGACGTACCTGAGAAAGAGGTTTCCACTGAGGACGAAGCACAGACAGAAAGCGGACGCAAGAACGCTCGTGCTGGCGGTAAGAAATCCTTGAGTGTAGCCCGTAg                                                                                                                                           >  1:218187/1‑301 (MQ=255)`  
                                                                                                                                                                          `aCTTTAAGGAGGTATAAGTTATGGGTAAGAAAGTTAAGAAGGCCGTGAAGAAAGTCACCAAGTCTGTTAAGAAAGTCGTTAAGGAAGGGGCTCGTCCGGTTAAACAGGTTGCTGGC‑‑‑‑‑‑‑‑‑GGTCTGGCTGGTGGTACTGGTGAAGCACAGATGGTGGAAGTACCACAAGCTGCCGCACAGATTGTTGACGTACCTGAGAAAGAGGTTTCCACTGAGGACGAAGCACAGACAGAAATCGGACGCAAGAAAGCTCGTGCTGGCGGTAAGAAATCCTTGAGTGTAGCCCGTAGCTCCGGTgctgtctt                                                                                                                            >  1:212573/1‑294 (MQ=255)`  
                                                                                                                                                                          `aCTTTAAGGAGGTATAAGTTATGGGTAAGAAAGTTAAGAAGGCCGTGAAGAAAGTCACCAAGTCCGTTAAGAAAGTCGTTAAGGAAGGGGCTCGTCCGGTTAAACAGGTTGCTGGCGGTCTAGCTGGTCTGGCTGGTGGTACTGGTGAAGCACAGATGGTGGAAGTACCACAAGCTGCCGCACAGATTGTTGACGTACCTGAGAAAGAGGTTTCCACTGAGGACGAAGCACAGACAGAAAGCGGACGCAAGAAAGCTCGTGCTGGCGGTAAGAAATCCTTGAGTGTAGCCCGTAGCTCCgg                                                                                                                                     >  1:520228/1‑301 (MQ=255)`  
                                                                                                                                                                          `aCTTTAAGGAGGTATAAGTTATGGGTAAGAAAGTTAAGAAGGCCGTGAAGAAAGTCACCAAGTCCGTTAAGAAAGTCGATAAGGAAGGGGCTCGTCCGGTTAAACAGGTTGCTGGCGGTCTAGCTGGTCTGGCTGGTGGTACTGGTGAAGCACAGATGGTGGAAGTACCACAAGCTGCCGCACAGATTGTTGACGTACCTGAGAAAGAGGTTTCCACTGAGGACGAAGCACAGACAGAAAGCGGACGCAAGAAAGCTCGTGCTGGCGGTAAGAAATCCTTGAGTGTAGCCCGTAGCTCCgg                                                                                                                                     >  1:93927/1‑301 (MQ=255)`  
                                                                                                                                                                           `cTTTAATGAGGTATAAGTTATGGGTAAGAAAGTTAAGAAGGCCGTGAAGAAAGTCACCAAGTCCGTTAAGAAAGTCGTTAAGGAAGGGGCTCGTCCGGTTAAACAGGTTGCTGGCGGTCTAGCTGGTCTGGCTGGTGGTACTGGTGAAGCACAGATGGTGGAAGTACCACAAGCTGCCGCACAGATTGTTGACGTACCTGAGAAAGAGGTTTCCACTGAGGACGAAGCACAGACAGAAAGCGGACGCAAGAAAGCTCGTGCTGGCGGTACGAAATCCTTGAGTGTAGCCCGTAGCTCCggg                                                                                                                                    >  1:424645/1‑300 (MQ=255)`  
                                                                                                                                                                           `cTTTAAGTAGGTATAAGTTATGGGTAAGAAAGTTAAGAAGGCCGTGAAGAAAGTCACCAAGTCCGTTAAGAAAGTCGTTAAGGAAGGGGCTCGTCCGGTTAAACAGGTTGCTGGCGGTCTAGCTGGTCTGGCTGGTGGTACTGGTGAAGCACAGATGGTGGAAGTACCACAAGCTGCCGCACAGATTGTTGACGTACCTGAGAAAGAGGTTTCCACTGAGGACGAAGCACAGACAGAAAGCGGACGCAAGAAAGCTCGTGCTGGCGGTAAGAAATCCTTGGGTGTAGcccctgtctcttat                                                                                                                                    >  1:617440/1‑290 (MQ=255)`  
                                                                                                                                                                           `cTTTAAGGAGGTATAAGTTATGGGTAAGAAAGTTAAGAAGGCCGTGAAGAAAGTCACCAAGTCCGTTAAGAAAGTCGTTAAGGAAGGGGCTCGTCCGGTTAAACAGGTTGCTGGCGGTCTAGCTGGTCTGGCTGGTGGTACTGGTGAAGCACAGATGGTGGAAGTACCCCAAGCTGCCGCACAGTTTGTTGACGTACCTGAGAAAGAGGTTTCCACTGAGGACGAAGAACAGACAGAAAGCGGAAGCAAGAAAGCTCGTGCTGGCGGTAGGAAATCCTTGAGTGTAGCCGGTAGCTCCggg                                                                                                                                    >  1:335874/1‑300 (MQ=255)`  
                                                                                                                                                                                  `gaggTATAAGTTATGGGTAAGAAAGTTAAGAAGGCCGTGACGAAAGTCACCAAGTCCGTTAAGAAAGTCGTTAAGGAAGGGGCTCGTCCGGTTAAACAGGTTGCTGGCGGTCTAGCTGGTCTGGCTGGTGGTACTGGTGAAGCACAGATGGTGGAAGTACCACAAGCTGCCGCACAGATTGTTGACGTACCTGAGAACGAGGTTTCCACTGAGGACGAAGCACAGACAGAAAGCGGACGCAAGAAAGCTCGTGCTGGCGGTACGAAATCCTTGAGTGTAGCCCGTAGCTCCGGTGGCGGtc                                                                                                                             >  1:508931/1‑300 (MQ=255)`  
                                                                                                                                                                                    `ggTATAAGTTATGGGTAAGAAAGTTAAGAAGGCCGTGAAGAAAGTCACCAAGTCCGTTAAGGAAGTCGTTAAGGAAGGGGATCGTCCGGTTAAACAGGTTGCTGGCGGTCTAGCTGGTCTGGCTGGTGGTACTGGTGAAGCACAGATGGTGGAAGTACCACAAGCTGCTGCACAGATTGTTGACGTACCTGAGAAAGAGGTTTCCACTGAGGACGAAGCACAGACAGAAAGCGGACGCAAGAAAGCTCGTGCTGGCGGTAAGAAATCCTTGAGTGTAGCCCGTAGCTCCGGTGGCGGTATc                                                                                                                           >  1:436401/1‑301 (MQ=255)`  
                                                                                                                                                                                    `ggTATAAGTTATGGGTAAGAAAGTTAAGAAGGCCGTGAAGAAAGTCACCAAGTCCGTTAAGAAAGTCGTTAAGTAAGGGGCTCGTCCGGTTAACCAGGTTGCTGGCGGTCTAGCTGGTCTGGCTGGTGGTACTGGTGAAGCACAGATGGTGGAAGTACCACAAGCTGCCGCACAGATTGTTGACGTACCTGAGAAAGAGGTTTCCACTGAGGACGAAGCACAGACAGAAAGCGGACGCAAGAAAGCTCGTGCTGGCGGTAAGAAATCCTTGAGTGTAGCCCGTAGCTCCGGTGGCGGTATc                                                                                                                           >  1:429707/1‑301 (MQ=255)`  
                                                                                                                                                                                    `ggTATAAGTTATGGGTAAGAAAGTTAAGAAGGCCGTGAAGAAAGTCACCAAGTCCGTTAAGAAAGTCGTTAAGGAAGGGGCTCGTCCGGTTAAACAGGTTGCTGGCGGTCTAGCTGGTCTGGCTGGTGGTACTGGTGAAGCACAGATGGTGGAAGTACCACAAGCTGCCGCTCAGATTGTTGACGTACCTGAGAAAGAGGTTTCCACTGAGGACGAAGCACAGACAGAAAGCGGACGCAAGAAAGCTCGTGCTGGCGGTAAGAAATCCTTGAGTGTAGCCCGTAGCTCCGGTGGCGGTATc                                                                                                                           >  1:132183/1‑301 (MQ=255)`  
                                                                                                                                                                                    `ggTATAAGTTATGGGTAAGAAAGTTAAGAAGGCCGTGAAGAAAGTCACCAAGTCCGTTAAGAAAGTCGTTAAGGAAGGGGCTCGTCCGGTTAAACAGGTTGCTGGCGGTCTAGCTGGTCTGGCTGGTGGTACTGGTGAAGCACAGATGGTGGAAGTACCACAAGCTGCCGCACAGATTGTTGACGTACCTGAGAAAGAGGTTTCCACTGATGACGAAGCACAGACAGAAAGCGGACGCAAGAAAGCTCGTGCTGGCGGTAAGAAATCCTTGAGTGTAGCCCGTAGCTCCGGTGGCGGTATc                                                                                                                           >  1:609455/1‑301 (MQ=255)`  
                                                                                                                                                                                    `ggTATAAGTTATGGGTAAGAAAGTTAAGAAGGCCGTGAAGAAAGTCACCAAGTCCGTTAAGAAAGTCGTTAAGGAAGGGGCTCGTCCGGTTAAACAGGTTGCTGGCGGTCTAGCTGGTCTGGCTGGTGGTACTGGTGAAGCACAGATGGTGGAAGTACCACAAGCTGCCGCACAGATTGTTGACGTACCTGAGAAAGAGGTTTCCACTGAGGACGAAGCACAGACAGAAAGCGGCCGCAAGAAAGCTCGTGCTGGCGGTAAGAAATCCTTGAGTGTAGCCCGTAGCTCCGGTGGCGGTATc                                                                                                                           >  1:652228/1‑301 (MQ=255)`  
                                                                                                                                                                                     `gTATAAGTTATGGGTAAGAAAGTTAAGAAGGCCGTGAAGAAAGTCACCAAGTCCGTTAAGCAAGTCGTTAAGGAAGGGGCTCGTCCGGTTAAACAGGTTGCTGGCGGTCTAGCTGGTCTGGCTGGTGGTACTGGTGAAGCACAGATGGTGGAAGTACCACAAGCTGCCGCACAGATTGTTGACGTACCTGAGAAAGAGGTTTCCACTGAGGACGAAGCACAGACAGAAAGCGAACGCAAGAAAGATCGTGCCGGCGGTAGGAAATCCTTGAGTGTAGCCCGTAGcctgtcgcttatacaca                                                                                                                          >  1:181185/1‑285 (MQ=255)`  
                                                                                                                                                                                     `gTATAAGTTATGGGTAAGAAAGTTAAGAAAGCCGTGAAGAAAGTCACCAAGTCCGTTAAGAAAGTCGTTAAGGAAGGGGCTCGTCCGGTTAAACAGGTTGCTGGCGGTCTAGCTGGTCTGGCTGGTGGTACTGGTGAAGCACAGATGGTGGAAGTACCACAAGCTGACGCACAGATTGTTGACGTACCGGAGAAAGAGGTTTCCACTGAGGACGAAGCACAGACAGAAAGCGGACGCAAGAAAGCTCGTGCTGGCGGTAAGAAATCCTTGAGTGTAGACCGTAGCTCCGGTGGCGGTATCa                                                                                                                          >  1:353550/1‑301 (MQ=255)`  
                                                                                                                                                                                         `aaGTGATGGGGAAGAAAGTTAAGAAGGCCGTGAAGAAAGTCACACAGTCCGTTAAGAACGTCGTTAAGGAAGGGGCTCGTCCGGTTAAACAGGTTGCTGGCGGTCTAGCTGGTCTGGCTGGTGGTACTGGTGAAGAACAGATGGTGGAAGTACCACAAGCTGCCGCACAGATTGTTGACGTACCTGAGAAAGAGGTTTCCACTGAGGACGAAGCACAGACAGAAAGCGGACGCAAGAAAGCTTGTGCTGGCTGTAAGAAATCCTTGAGTGTAGCCCGTAGCTCCGGTGGCGGTATCAACAt                                                                                                                      <  1:241317/301‑1 (MQ=255)`  
                                                                                                                                                                                          `aGTTATTGGTAAGAAAGTTAAGAAGGCCGTGAAGAAAGTCACCAAGTCCGTTAAGAAAGTCGTTAAGGAAGGGGCTCGTCCGGTTAAACAGGTTGCTGGCGGTCTAGCTGGTCTGGCTGGTGGTACTGGTGAAGCACAGATGGTGGAAGTACCACAAGCTGCCGCACAGATTGTTGACGTACCTGAGAAAGAGGTTTCCACTGAGGACGAAGCACAGACAGAAAGCGGACGCAAGAAAGCTCGTGCTGGCGGTAAGAAATCCTTGAGTGTAGCCCGTAGCTCCGGTGGCGGTATCAACAtt                                                                                                                     >  1:100073/1‑301 (MQ=255)`  
                                                                                                                                                                                                               `gaagGCCGTGAAGAAAGTCACCAAGTCCGTTAAGAAAGTCGTTAAGGAAGGGGCTCGTCCGGTTAAACAGGTTGCTGGCGGTCTAGCTGGTCTGGCTGGTGGTACTGGTGAAGCACAGATGGTGGAAGTACCACAAGCTGCCGCACAGATTGTTGACGTACCTGAGAAAGAGGTTTCCACTGAGGACGAAGCACAGACAGAAAGCGGACGCAAGAAAGCTCGTGCTGGCGGTACGAAATCCTTGAGTGTAGCCCGTAGCTCCGGTGGCGGTATCAACATTTAATCAGGAGGTTATCGTGGa                                                                                                >  1:473843/1‑301 (MQ=255)`  
                                                                                                                                                                                                                          `agaaAGTCACCAAGTCCGTTAAGAAAGTCGTTAAGGAAGGGGCTCGTCCGGTTAAACAGGTTGCTGGCGGTCTAGCTGGTCTGGCTGGTGGTACTGGTGAAGCACAGATGGTGGAAGTACCACAAGCTGCCGCACAGATTGTTGACGTACCTGAGAAAGAGGTTTCCACTGAGGACGAAGCACAGACAGAAAGCGGACGCAAGAAAGCTCGTGCTGGCGGTAATAAATCCTTTAGTGTAGCCCGTAGCTCCGGTGGCGGTATCAACATTTAATCAGGAGGTTATCGTAGAAGACTGCATTg                                                                                     <  1:663525/301‑1 (MQ=255)`  
                                                                                                                                                                                                                             `tgtgtataagagacagtAAGAAAGTCGTTAAGGAAGGGGCTCGTCCGGTTAAACAGGTTGCTGGCGGTCTAGCTGGTCTGGCTGGTGGTACTGGTGAAGCACAGATGGTGGAAGTACCACAAGCTGCCGCACAGATTGTTGACGTACCTGAGAAAGAGGTTTCCACTGAGGACGAAGCACAGACAGAAAGCGGACGCAAGAAAGCTCGTGCTGGCGGTAAGAAATCCTTGAGTGTAGCCCGTAGCTCCGGTGGCGGTATCAACATTTAATCAGGAGGTTATCGTGGAAGACTGCATTGAAt                                                                                  <  1:341229/285‑1 (MQ=255)`  
                                                                                                                                                                                                                                 `cACCAAGTCCGTTAAGAAAGTCGTTAAGGAAGGGGCTCGTCCGGTTAAACAGGTTGCTGGCGGTCTAGCTGGTCTGGCTGGTGGTACTGGTGAAGCACAGATGGTGGAAGTACCACAAGCTGCCGCACAGATTGTTGACGTACCTGAGAAAGAGGTTTCCACTGAGGACGAAGCACAGACAGAAAGCGGACGCAAGAAAGCTCGTGCTGGCGGTAAGAAATCCTTGAGTGTAGCCCGTAGCTCCGGTGGCGGTATCAACATTTAATCAGGAGGTTATCGTGGAAGACTGCATTGAATGGAc                                                                              >  1:646990/1‑301 (MQ=255)`  
                                                                                                                                                                                                                                  `gatgtgtataagagacaggTCGTTAAGGAAGGGGCTCGTCCGGTTAAACAGGTTGCTGGCGGTCTAGCTGGTCTGGCTGGTGGTACTGGTGAAGCACAGATGGTGGAAGTACCACAAGCTGCCGCACAGATTGTTGACGTACCTGAGAAAGAGGTTTCCACTGAGGACGAAGCACAGACAGAAAGCGGACGCAAGAAAGCTCGTGCTGGCGGTAAGAAATCCTTGAGTGTAGCCCGTAGCTCCGGTGGCGGTATCAACATTTAATCAGGAGGTTATCGTGTAAGACTGCATTGAATGGAcc                                                                             <  1:223122/283‑1 (MQ=255)`  
                                                                                                                                                                                                                                    `cAAGTCCGTTAAGAAAGGCGTTAAGGAAGGGGTTCGTACGGTTAAAAAGGTAGCGGGCGGTCTAGCTGGTCTGGCAGGTGGTACTGGTGAAGCAAAGATGGTGGAAGTACCAGAAGCTGCCGCACAGATTGTTGACGTACCTGAGAAAGAGGTTACCACTGAGGACGAAGCACAGACAGAAAGCGGACGCAAGAAAGCTCGTGCTGGCGGTAAGAAATCCTTGAGTGTAGCCCGTAGCTCCGGTGGCGATATCAACATTTAATCAGGAGGTTATCGTGGAAGACTGCATTGAATGGACCgg                                                                           <  1:48441/301‑1 (MQ=255)`  
                                                                                                                                                                                                                                      `aGTCCGTTAAGAAAGTCGTTAAGGAAGGGGCTCGTCCGGTTAAACAGGTTGCTGGCGGTCTAGCTGGTCTGGCTGGTGGTACTGGTGAAGCACAGATGGTGGAAGTACCACAAGCTGCCGCACAGATTGTTGACGTACCTGAGAAAGAGGTTTCCACTGAGGACGAAGCACAGACAGAAAGCGGACGCAAGAAAGCTCGTGCTGGCGGTAAGAAATCCTTGAGTGTAGCCCGTAGCTCCGGTGGCGGTATCAACATTTAATCAGGAGGTTATCGTGGAAGACTGCATTGAATGGACCGGAg                                                                         >  1:634017/1‑301 (MQ=255)`  
                                                                                                                                                                                                                                               `aGATAGTCGTTTAGGAAGGGGCTCGTCCGGTTACACAGGTTGCTGGCGGTCTAGCTGGTCTGGCTGGTGGTACTGGTGAAGCACAGATGGTGGAAGTACCACAAGCTGCCGCACAGATTGTTGACGTACCTGAGAAAGAGGTTTCCACTGAGGACGAAGCACAGACAGAAAGCTGACGCAAGAAAGCTCGTGCTGGCGGTAAGAAATCCTTGAGTGTAGCCCGTAGCTCAGGTGGCGGTATCAACATTTAATCAGGAGGTTATCGTGGAAGACTGCATTGAATGGACCGGAGGTGTCAAct                                                                <  1:71418/301‑1 (MQ=255)`  
                                                                                                                                                                                                                                                 `aaaGTCGTTAAGGAAGGGGCTCGTCCGGTTAAACAGGTTGCTGGCGGTCTAGCTGGTCTGGCTGGTGGTACTGGTGAAGCACAGATGGTGGAAGTACCACAAGCTGCCGCACAGATTGTTGACGTACCTGAGAAAGAGGTTTCAACTGAGGACGAAGCACAGACAGAAAGCGGACGCAAGAAAGCTCGTGCTGGCGGTAAGAAATCCTTGAGTGTAGCCCGTAGCTCCGGTGGCGGTATCAACATTTAATCAGGAGGTTATCGTGGAAGACTGCATTGAATGGACCGGAGGTGTCAActct                                                              >  1:455144/1‑301 (MQ=255)`  
                                                                                                                                                                                                                                                  `aaGTCGTTAAGGAAGGGGCTCGTCCGGTTAAACAGGTTGCTGGCGGTCTAGCTGGTCTGGCTGGTGGTACTGGTGAAGCACAGATGGTGGAAGTACCACAAGCTGCCGCACAGATTGTTGACGTACCTGAGAAAGAGGTTTCCACTGAGGACGAAGCACAGACAGAAAGCGGACGCAAGAAAGCTCGTGCTGGCGGTAAGAAATCCTTGAGTGTAGCCCGTAGCTCCGGTGGCGGTATCAACATTTAATCAGGAGGTTATCGTGGAAGACTGCATTGAATGGACCGGAGGTGTCAACTCTa                                                             >  1:323334/1‑301 (MQ=255)`  
                                                                                                                                                                                                                                                     `tCGTTAAGGAAGGGGCTCGTCCGGTTAAACAGGTTGCTGGCGGTCTAGCTGGTCTGGCTGGTGGTACTGGTGAAGCACAGATGGTGGAAGTACCACAAGCTGCCGCACAGATTGTTGACGTACCTGAGAAAGAGGTTTCCACTGAGGACGAAGCACAGACAGAAAGCGGACGCAAGAAAGCTCGTGCTGGCGGTAAGAAATCCTTGAGTGTAGCCCGTAGCTCCGGTGGCGGTATCAACATTTAATCAGGAGGTTATCGTGGAAGACTGACTTGAATGGAGCGGAGGTGTCAACTCTaaag                                                          >  1:88588/1‑299 (MQ=255)`  
                                                                                                                                                                                                                                                      `cGTTAAGGAAGGGGCTCGTCCGGTTACACAGGTTGCTGGCGGTCTAGCTGGTCTGGCTGGTGGTACTGGTGAAGCACAGATGGTGGAAGTACCACAAGCTGCCGCACAGATTGTTGACGTACCTGAGAAAGAGGTTTCCACTGAGGACGAAGCACAGACAGAAAGCGGACGCAAGAAAGCTCGTGCTAGCGGTAAGAAATCCTTGAGTGTAGCCCGTAGCTCCGGTGGCGGTATCAACATTTAATCAGGAGGTTATCGTGGAAGACTGCATTGAATGGACCGGAGGTGTCAACTCTAAggg                                                         <  1:347343/301‑1 (MQ=255)`  
                                                                                                                                                                                                                                                      `cGTTAAGGAAGGGGCTCGTCCGGTTAAACAGGTTGCTGGCGGTCTAGCTGGTCTGGCTGGTGGTACTGGTGAAGCACAGATGGTGGAAGTACCACAAGCTGCCGCACAGATTGTTGACGTACCTGAGAAAGAGGTTTCCACTGAGGACGAAGCACAGACAGAAAGCGGACGCAAGAAAGCTCGTGCTGGCGGTAAGAAATCCTTGAGTGTAGCCCGTAGCTCCGGTGGCGGTATCAACATTTAATCAGGAGGTTATCGTGGAAGACTGCATTGAATGGACCGGAGGTGTCAACTCTAAggg                                                         <  1:306852/301‑1 (MQ=255)`  
                                                                                                                                                                                                                                                      `cGTTAAGGAAGGGGCTCGTCCGGTTAAACAGGTTGCTGGCGGTCTAGCTGGTATGGCTGGTGGTACTGGTGAAGCACAGATGGTGGAAGTACCACAAGCTGCCGCACAGATTGTTGACGTACCTGAGAAAGAGGTTTCCACTGAGGACGAAGCACAGACAGAAAGCGGACGCAAGAAAGCTCGTGCTGGCGGTAAGAAATCCTTGAGTGTAGCCCGTAGCTCCGGTGGCGGTATCAACATTTAATCAGGAGGTTATCGTGGAAGACTGCATTGAATGGACCGGAGGTGTCAACTCTAAggg                                                         <  1:549118/301‑1 (MQ=255)`  
                                                                                                                                                                                                                                                            `ggaaggGGTTCGTCCGGTTAAACAGGTTTCTGGCGGTCTAGCTGGTCTGGCTGGTGGTACTGGTGAAGCACAGATGGTGGAAGTACCACAAGATGCCGCACAGATTGTTGACGTACCTGAGAAAGAGGTTTCCACTGAGGACGAAGCACAGACAGAAAGCGGACGCAAGAAAGCTCGTGCTGGCGGTAAGAAATCCTTGAGTGTAGCCCGTAGCTCCGGTGGCGGTATCAACATTTAATCAGGAGGTTATCGTGGAAGACTGCATTGAATGGACAGGAGGTGTCAACTCTAAGGGTTATgg                                                   <  1:461690/301‑1 (MQ=255)`  
                                                                                                                                                                                                                                                            `ggaaggGGCTCGTCCGGTTAAACAGGTTGCTGGCGGTCTAGCTGGTCTGGCTGGTGGTACTGGTGAAGCACAGATGGTGGAAGTACCACAAGCTGCTGCACAGATTGTTGACGTACCTGAGAAAGAGGTTTCCACTGAGGACGAAGCACAGACAGAAAGCGGACGCAAGAAAGCTCGTGCTGGCGGTAAGAAATCCTTGAGTGTAGCCCGTAGCTCCGGTTGCGGTATCAACATTTAATCAGGAGGTTATCATGGAAGACTGCATTGAATGGACCGGAGGTGTCAACTCTAAGGGTTATgg                                                   >  1:270531/1‑301 (MQ=255)`  
                                                                                                                                                                                                                                                            `ggaaggGGCTCGTCCGGTTAAACAGGTTGCTGGCGGTCTAGCTGGTCTGGCTGGTGGTACTGGTGAAGCACAGATGGTGGAAGTACCACAAGCTGCCGCACAGATTGTTGACGTACCTGAGAAAGAGGTTTCCACTGAGGACGAAGCACAGACAGAAAGCGGATGCAAGAAAGCTCGTGCTGGCGGTAAGAAATCCTTGAGTGTAGCCCGTAGCTCCGGTGGCGGTATCAACATTTAATCAGGAGGTTATCGTGGAAGACTGCATTGAATGTACCGGAGGTGTCAACTCTAAGGGTTATgg                                                   <  1:282714/301‑1 (MQ=255)`  
                                                                                                                                                                                                                                                                  `ggCTCGTCCGGTTAAACAGGTTGCTGGCGGTCTAGCTGGTCTGGCTGGTGGTACTGGTGAAGCACAGATGGTGGAAGTACCACAAGCTGCCGCACAGATTGTTGACGTACCTGAGAAAGAGGTTTCCACTGAGGACGAAGCACAGACAGAAAGCGGACGCAAGAAAGCTCGTGCTGGCGGTAAGAAATCCTTGAGTGTAGCCCGTAGCTCCGGTGGCGGTATCAACATTTAATCAGGAGGTTATCGTGGAAGACTGCATTGAATGGACCGGAGGTGTCAACTCTAAgggctgtctcttata                                             >  1:228830/1‑289 (MQ=255)`  
                                                                                                                                                                                                                                                                   `gcacGTCCGGTTAAACAGGGTGCTGGCGGTCTAGCTGGTCTGGCTGGTGGTACTGGGGAAGCACAGATGGTGGAAGTAACACAAGCTGCCGCACAGATTGTTGACGTACCTGAGAAAGAGGTTTCCACTGAGGACGAAGCACAGACAGAAAGCGGACGCAAGAAAGCTCGTGCTGGCGGTAAGAAATCCTTGAGTGTAGGCCGTAGCTCCGGTGGCGGTATCAACATTAAATCAGGAGGTTATCGTGGAAGACTGCATTGAATGGACCGGAGGTGTCAACTCTAAGGGTTATGGTCGTAAg                                            <  1:106174/298‑1 (MQ=255)`  
                                                                                                                                                                                                                                                                   `gCTCGTCCGGTTAAACAGGTTGCTGGCGGTCTAGCTGGTCTGGCTGGTGGTACTGGTGAAGCACAGATGGTGGAAGTACCACAAGCTGCCGCACAGATTGTTGACGTACCTGAGAAAGAGGTTTCCACTGAGGACGAAGCACAGACAGAAAGCGGACGCAAGAAAGCTCGTGCTGGCGGTAAGAAATCCTTGAGTGTAGCCCGTAGCTACGGTGGCGGTATCAACATTTAATCAGGAGGTTATCGTGGAAGACTGCATTGAATGGACCAGAGGTGTCAACTCTAAGGGTTATGGTCGTAAg                                            <  1:246083/301‑1 (MQ=255)`  
                                                                                                                                                                                                                                                                   `gCTCGTCCGGTTAAACAGGTTGCTGGCGGTCTAGCTGGTCTGGCTGGTGGTACTGGTGAAGCACAGATGGTGGAAGTACCACAAGCTGCCGCACAGATTGTTGACGTACCTGAGAAAGAGGTTTCCACGGAGGACGAAGCACAGACAGAAAGCGGACGCAAGAAAGCTCGTGCTGGCTGTAAGAAATCCTTGAGTGTAGCCCGTAGCTCCGGTGGCGGTATCAACATTTAATCAGGAGGTTATCGTGGAAGACTGCATTGAATGGACCGGAGGTGTCAACTCTAAGGGTTATGGTCGTAAg                                            <  1:126679/301‑1 (MQ=255)`  
                                                                                                                                                                                                                                                                    `cTCGTCCGGTTAAACAGGTTGCTGGCGGTCTAGCTGGTCTGGCTGGTGGTACTGGTGAAGCACAGAGGGTGGAAGTACCACAAGCTGCCGCACAGATTGTTGACGTACCTGAGAAAGAGGTTTCCACTGAGGACGAAGCACAGACAGAAAGCGGACGCAAGAAAGCTCGTGCTGGCGGTAAGAAATCCTTGAGTGTAGCCCGTAGCTCCGGTGGCGGTATCAACATTTAATCAGTAGGTTATCGTGGAAGACTGCATTGAATGGACCGGAGGTGTCAACTCTAAGGGTTATGGTCGTAAGt                                           <  1:371283/301‑1 (MQ=255)`  
                                                                                                                                                                                                                                                                        `tCCGGTTAAACAGGTTGCTGGCGGTCTAGCTGGTCTGGCTGGTGGTACTGGTGAAGCACAGATGGTGGAAGTACCACAAGCTGCCGCACAGATTGTTGACGTACCTGAGAAAGAGGTTTCCACTGAGGACGAAGCACAGACAGAAAGCGGACGCAAGAAAGCTCGTGCTGGCGGTAAGAAATCCTTGAGTGTAGCCCGTAGCTCCGGTGGCGGTATCAACATTTAATCAGGAGGTTATCGTGGAAGACTGCATTGAATGGACCGGAGGTGTCAACTCTAAGGGTTATGGTCGTAAGTGGGt                                       <  1:467226/301‑1 (MQ=255)`  
                                                                                                                                                                                                                                                                              `tAAACAGGTTGCTGGCGGTCTAGCTGGTCTGGCTGGTGGTACTGGTGAAGCACAGATGGTGGAAGTACCACAAGCTGCCGCACAGATTGTTGACGTACCTGAGAAAGAGGTTTCCACTGAGGACGAAGCACAGACAGAAAGCGGACGCAAGAAAGCTCGTGCTGGCGGTAAGAAATCCTTGAGTGTAGCCCGTAGCTCCGGTGGCGGTATCAACATTTAATCAGGAGGTTATCGTGGAAGACTGCATTGAATGGACCGGAGGTGTCAACTCTAAGGGTTATGGTCGTAAGTGGGTTAATgg                                 <  1:295317/301‑1 (MQ=255)`  
                                                                                                                                                                                                                                                                                    `gggtGCTGGCGGTCTAGCTGGTCTTGCTGGTGGTACTGGTGAAGCACAGATGGTGGAAGTACCACAAGCTGCCGCACAGATTGTTGAAGTACCTGAGAAAGAGGTTTCCACTGAGGACGAAGCACAGACAGAAAGCGGACGCAAGAAAGCTCGTGCTGGCGGTAAGAAATCCTTGAGTGTAGCCCGTAGCTCCGGTGGCGGTATCAACATTTAATCAGGAGGTTATCGTGGAAGACTGCATTGAATGGACCGGAGGTGTCAACTCTAAGGGTTATGGTCGTAAGTGGGTTAATGGTAAACt                           <  1:577227/298‑1 (MQ=255)`  
                                                                                                                                                                                                                                                                                          `tGGCGGTCTAGCTGGTCTGGCTGGTGGTACTGGTGAAGCACAGATGGTGGCAGTACCACAAGCTGCCGCACAGATTGTTGACGTACCTGAGAAAGAGGTTTCCACTGAGGACGAAGCACAGACAGAAAGCGGACGCAAGAAAGCTCGTGCTGGCGGTAAGAAATCCTTGAGTGTAGCCCGTAGCTCCGGTGGCGGTATCAACATTTAATCAGGAGGTTATCGTGGAAGACTGCATTGAATGGACCGGAGGTGTCAACTCTAAGGGTTATGGTCGTAAGTGGGTTAATGGTAAACTTGTGAc                     <  1:143627/301‑1 (MQ=255)`  
                                                                                                                                                                                                                                                                                                       `ggTCTGGCTGGTGGTACTGGTGAAGCACAGATGGTGGAAGTACCACAAGCTGCCGCACAGATTGTTGACGTACCTGAGAAAGAGGTTTCCACTGAGGACGAAGCACAGACAGAAAGCGGACGCAAGAAAGCTCGTGCTGGCGGTAAGAAATCCTTGAGTGTAGCCCGTAGCTCCGGTGGCGGTATCAACATTTAATCAGGAGGTTATCGTGGAAGACTGCATTGAATGGACCGGAGGTGTCAACTCTAAGGGTTATGGTCGTAAGTGGGTTAATGGTAAACTTGTGACTCCACATAGGcac        >  1:318962/1‑301 (MQ=255)`  
                                                                                                                                                                                                                                                                                                        `gTCTGGCTGGTGGTACTGGTGAAGCACAGATGGTGGAAGTACCACAAGCTGCCGCACAGATTGTTGACGTACCTGAGAAAGAGGTTTCCACTGAGGACGAAGCACAGACAGAAAGCGGACGCAAGAAAGCTCGTGCTGGCGGTAAGAAATCCTTGAGTGTAGCCCGTAGCTCCGGTGGCGGTATCAACATTTAATCAGGAGGTTATCGTGGAAGACTGCATTGAATGAACCGGAGGTGTCAACTCTAAGGGTTATGGTCGTAAGTGGGTTAATGGGAAACTTGTGAcctgtctcttataca       >  1:377350/1‑287 (MQ=255)`  
                                                                                                                                                                                                                                                                                                            `ggctggTGGTACTGGTGAAGCACAGATGGTGGAAGTACCACAAGCTGCCGCACAGATTGTTGACGTACCTGAGAAAGAGGTTTCCACTGAGGACGAAGCACAGACAGAAAGCGGACGCAAGAAAGCTCGTGCTGGCGGTAAGAAATCCTTGAGTGTAGCCCGTAGCTCCGGTGGCGGTATCAACATTTAATCAGGAGGTTATCGTGGAAGACTGCATTGAATGGACCGGAGGTGTCAACTCTAAGGGTTATGGTCGTAAGTGGGTTAATGGTAAACTTGTGACTCCACATAGGCACATCtc   >  1:623166/1‑300 (MQ=255)`  
                                                                                                                                                                                                                                                                                                            `ggctggTGGTACTGGTGAAGCACAGATGGTGGAAGTACCACAAGCTGCCGCACAGATTGTTGACGTACCTGAGAAAGAGGTTTCCACTGAGGACGAAGCACAGACAGAAAGCGGACGCAAGAAAGCTCGTGCTGGCGGTAAGAAATCCTTGAGTGTAGCCCGTAGCTCCGGTGGCGGTATCAACATTTAATCAGGAGGTTATCGTGGAAGACTGCATTGAATGGACCGGAGGTGTCAACTCTAAGGGTTATGGTCGTAAGTGGGTTAATGGTAAACTTGTGACTCCACATAGGCACATCTa   >  1:169576/1‑301 (MQ=255)`  

                                                                                                                                                                                                                                                                                                            `|`                                                                                                                                                                                                                                                                                                               
`CATTCACCACAAAGGAGACATTCTGGATGTAACGTCCGTTGTCATTAATCCTGAGGCAGACTCTAAGGGCTTACAGCGATTCCTAGCGAAACGCTTTAAGTACCTTGCGGAACTCCACGATTGCGATTGGGTGTCTCGTTGTAAGCATGAAGGCGAGACAATGCGTGTATACTTTAAGGAGGTATAAGTTATGGGTAAGAAAGTTAAGAAGGCCGTGAAGAAAGTCACCAAGTCCGTTAAGAAAGTCGTTAAGGAAGGGGCTCGTCCGGTTAAACAGGTTGCTGGCGGTCTAGCTGGTCTGGCTGGTGGTACTGGTGAAGCACAGATGGTGGAAGTACCACAAGCTGCCGCACAGATTGTTGACGTACCTGAGAAAGAGGTTTCCACTGAGGACGAAGCACAGACAGAAAGCGGACGCAAGAAAGCTCGTGCTGGCGGTAAGAAATCCTTGAGTGTAGCCCGTAGCTCCGGTGGCGGTATCAACATTTAATCAGGAGGTTATCGTGGAAGACTGCATTGAATGGACCGGAGGTGTCAACTCTAAGGGTTATGGTCGTAAGTGGGTTAATGGTAAACTTGTGACTCCACATAGGCACATCTAT  >  NC_001604.1/19345‑19946`

  

Alignment Legend

Aligned base mismatch/match (shaded by quality score): `ATCG/ATCG` `< 3 ≤` `ATCG/ATCG` `< 10 ≤` `ATCG/ATCG` `< 22 ≤` `ATCG/ATCG` `< 34 ≤` `ATCG/ATCG` `< 38 ≤` `ATCG/ATCG`

Unaligned base: `atcg`    Masked matching base: `atcg`    Alignment gap: `‑`    Deleted base: `‑`

Reads not counted as support for junction

read\_name Not counted due to insufficient overlap past the breakpoint.

read\_name Not counted due to not crossing MOB target site duplication.
